# ATP Citrate Lyase *ClACLB-1* Facilitates Citrate Cleavage in Lemon

**DOI:** 10.3390/plants14010053

**Published:** 2024-12-27

**Authors:** Chuang Lu, Wenhui Yang, Huaxi Zhang, Yanrong Wu, Huina Meng, Lifeng Lv, Wanping Lu, Dongmei Zhao, Guixiang Huang

**Affiliations:** College of Agriculture, Guangxi University, Nanning 530004, China; lc161804217@163.com (C.L.); yangwh1223@163.com (W.Y.); zhanghuaxi63@163.com (H.Z.); 17878773652@163.com (Y.W.); n2394828196@163.com (H.M.); lf1098297753@163.com (L.L.); luwp0117@163.com (W.L.); z320619533@126.com (D.Z.)

**Keywords:** lemon, citric acid, genome-wide identification, *ClACLB-1*, gene function

## Abstract

Citric acid is an important organic acid with wide applications and diverse biological functionality. As the predominant organic acid in lemons, citric acid plays a crucial role in determining the flavor of citrus, especially in lemons. ATP citrate lyase (ACL, EC4.1.3.8) is the keg gene in citric acid metabolism. Several research studies on ACL only focused on high-sugar- and low-acid-content citrus varieties; however, the ACL mechanism in lemons with high acid and low sugar levels remains undetermined. In this study, a key candidate gene, *ClACLB-1*, for citrate cleavage was identified from the genome data of ‘Xiangshui’ lemon [*Citrus limon* (L.) Burm f.]. The putative protein coded by the gene *ClACLB*-1 is localized in the nuclear and cell membranes. The *ClACLB*-1 gene was expressed in all tissues, with the highest expression in male flowers and the lowest expression in mature fruits; the expression decreased during lemon fruit development. The overexpression of *ClACLB*-1 in transgenic tomatoes significantly increases the activity of citrate lyase, which subsequently reduces citric acid content. This study clarified the function of the *ClACLB-1* gene in cleaving citric acid, provided new insights into the citric acid metabolism of citrus, and offered a theoretical reference for reducing acid and increasing sugar in citrus to improve fruit quality. It also helped to enhance the understanding of the metabolism and role of citrate in plants.

## 1. Introduction

Citric acid, an important organic acid, has a good antioxidant effect and unique advantages in reducing blood pressure, lowering blood sugar, and preventing stones and anti-aging [1,2,3,4]. In production, citric acid is widely used as an acidity regulator, flavoring agent, and chelating agent [5,6]. Citric acid not only is an essential organic acid in plant cell metabolism but also plays an important role in plant growth and development [7,8]. When plants are contaminated with heavy metals, citric acid and calcium work together to effectively alleviate the harm of heavy metals to plants. At the same time, by reducing oxidative damage and enhancing photosynthesis, plants maintain a good growth trend [9,10].

Citric acid is the main organic acid in citrus and a key component of fruit quality. The synthesis of citric acid involves phosphoenolpyruvate carboxylase (PEPC) carboxylating phosphoenolpyruvate (PEP) to form oxaloacetate (OAA), and acetyl-CoA formed by the oxidation of PEP is catalyzed by citrate synthase (CS) to form citric acid, which is then transported and stored in the vacuoles of plant cells [11,12,13,14]. In most citrus fruits, the content of citric acid decreases after ripening. However, in lemons, the content of citric acid increases with the ripening of fruits. There are many reasons for this phenomenon, such as fruit volume, water absorption, the activities of enzymes related to citrate cleavage, and the activities of enzymes related to synthesis [15]. The citric acid metabolism process in citrus fruits is very complex. At present, researchers generally believe that citric acid is synthesized in mitochondria and then accumulates in vacuoles. In the later stage of cell development, citric acid will be released from vacuoles and finally degraded in the cytoplasm [16]. Citric acid is mainly cleaved by ACL (citrate lyase) and ACO (aconitase). Citric acid can be cleaved into oxaloacetate and acetyl-CoA by ACL. Oxaloacetate and acetyl-CoA can re-enter the TCA or enter the gluconeogenesis or lipid metabolism pathways under the catalysis of many other enzymes [17]. ACO can catalyze citric acid to form isocitric acid, and isocitric acid can be further catalyzed to form α-ketoglutaric acid. At this stage, α-ketoglutaric acid can participate in the TCA cycle again or be catalyzed to form glutamic acid, and glutamic acid can continue to be catalyzed and enter γ-aminobutyric acid metabolism [18]. Extensive research confirmed that the citric acid degradation pathway in which ACO cleaves citric acid and finally makes glutamic acid enter the γ-aminobutyric acid metabolism plays a crucial role in the development of citrus fruits [18,19,20]. In a study on sweet lemons, it was found that the activation of the pathway in which ACO cleaves citric acid may be the reason for the decrease in citric acid [21]. In addition, some studies also showed that both the hot-air treatment of citrus fruits and exogenous application of GABA can activate the pathway in which ACO cleaves citric acid, resulting in significant changes in the content of citric acid in citrus fruits [22].

Oxaloacetate and acetyl-CoA are the products generated by citric acid in the ACL pathway. In a study on Clementine mandarin, it was first pointed out that there is a significant correlation between the decrease in the expression level of the *ACL* gene and the accumulation of citric acid during the fruit development of Clementine mandarin [18], and the expression level of this gene continues to decrease with fruit development. However, in a study on Satsuma mandarin, it was found that in the later stage of fruit development, the expression levels of the three genes encoding ACL increased significantly [23]. Some studies conducted a comparative analysis among six different varieties of citrus, and the results showed that there are obvious differences in the expression change patterns of the ACL gene among different varieties [24]. The content of citric acid in Red Anliu orange fruits is significantly lower than that in Anliu orange fruits, but the expression levels of *ACLA-1*, *ACLA-2*, and *ACLB* in Red Anliu orange fruits are several times higher than those in Anliu orange [25]. In general, ACL is closely related to citric acid metabolism. However, all existing studies on ACL focused on citrus varieties with high sugar and low acid content, and there is no research on lemons with high acid and low sugar content. Therefore, this article focused on the *ClACLB-1* gene in lemons, aiming to clarify the regulation of the *ClACLB-1* gene on citric acid metabolism, elucidate the relevant molecular mechanisms, and provide theoretical guidance for cultivating excellent lemon varieties.

## 2. Results

### 2.1. Identification of the ClACLB-1 Gene and Analysis of Conserved Motifs and Conserved Domains

**Figure 1:**
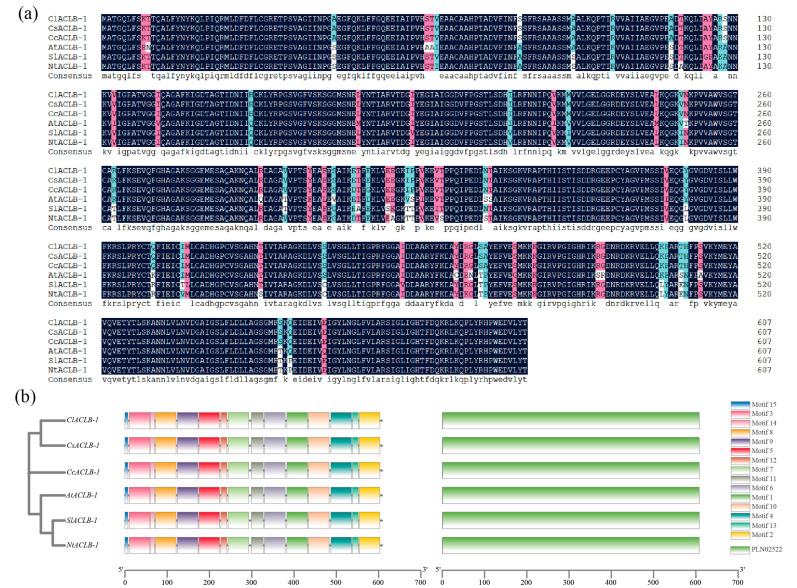
Multiple-sequence alignment, prediction of conserved motifs, and conserved domains of *ClACLB-1* with other species. (**a**) The similarity between *ClACLB-1* and homologous proteins of other species was 96.82%. (**b**) *ClACLB-1* and homologous proteins of other species both contained 15 conserved mods and 1 conserved domain.

The sequence of *AtACLB-1* in *Arabidopsis thaliana* was obtained from the NCBI. Then, the genome of ‘Xiangshui’ lemon was blasted using the TBtools software and combined with the representative domain (PF00028), and the *ClACLB-1* gene of ‘Xiangshui’ lemon (gene ID: evm.TU.ctg136.234) was obtained. The genomes of *Citrus sinensis*, *Citrus clementina*, *Arabidopsis thaliana*, *Nicotiana attenuate*, and *Solanum lycopersicum* were obtained from the website of citrus genomes (http://citrus.hzau.edu.cn/download.php (accessed on 16 November 2023)) and the website of plant genomes (https://plants.ensembl.org/info/data/ftp/index.html (accessed on 16 November 2023)). From these, the homologous genes of the *ClACLB-1* gene were identified, and their gene IDs were Cs9g02230.2, Ciclev10004572m.1, AT3G06650.1, OIS98573, and Solyc12g099260.2.1, respectively. Sequence analysis showed that the full-length ORF sequence of the *ClACLB-1* gene was 1827 bp, encoding a putative protein of 608 AA, with a relative molecular mass of 65,954.70 kD and an isoelectric point of 7.58. No signal peptide was found in the ClACLB-1 protein. Protein sequence alignment showed that the ClACLB-1 protein of lemon had 96.82% similarity with proteins of other species (Figure 1a). The ACLB-1 proteins of these six plants all contained 15 conserved motifs and a conserved domain named PLN02522 (Figure 1b).

### 2.2. Analysis of the Phylogenetic Tree of the ClACLB-1 Protein

**Figure 2:**
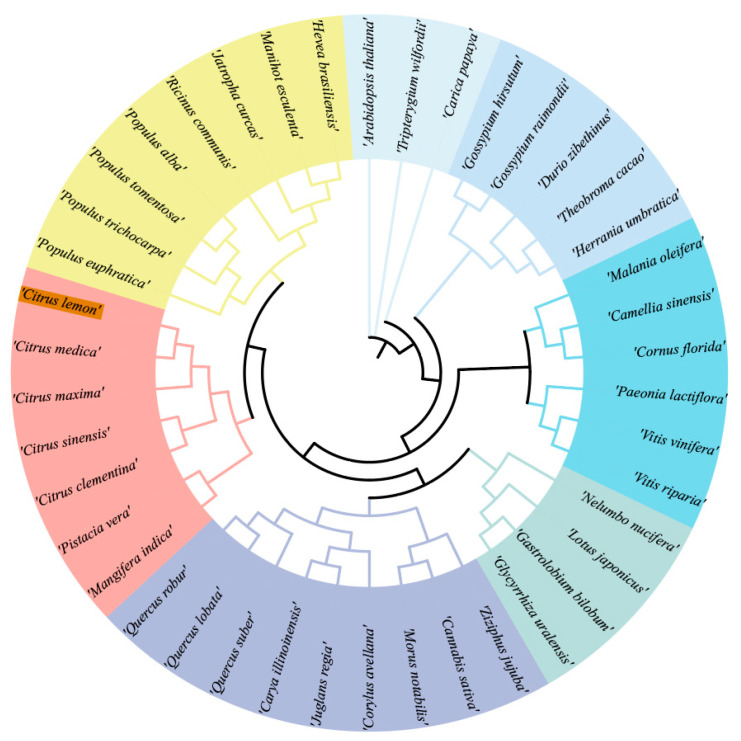
Phylogenetic evolutionary tree of ClACLB-1 protein of ‘Xiangshui’ lemon. The phylogenetic tree was divided into seven groups, represented by different colors, each of which was evolutionarily related to the species. The darker orange color indicates the ACLB-1 protein of ‘ Xiangshui‘lemon.

To analyze the phylogenetic relationship of the ClACLB-1 protein in ‘Xiangshui’ lemon, a phylogenetic tree analysis was performed using the amino acid sequence of *ClACLB-1* and homologous proteins from *Citrus sinensis*, *Citrus clementina*, *Arabidopsis thaliana*, and other species (Figure 2). The ClACLB-1 of ‘Xiangshui’ lemon has the closest relationship with *Citrus medica*, followed by *Citrus maxima*, *Citrus sinensis*, and *Citrus clementina*. Among other genera, it has a relatively close evolutionary relationship with *Mangifera indica*, *Pistacia vera*, and *Populus euphratica*.

### 2.3. Chromosomal Localization of the ClACLB-1 Gene

**Figure 3:**
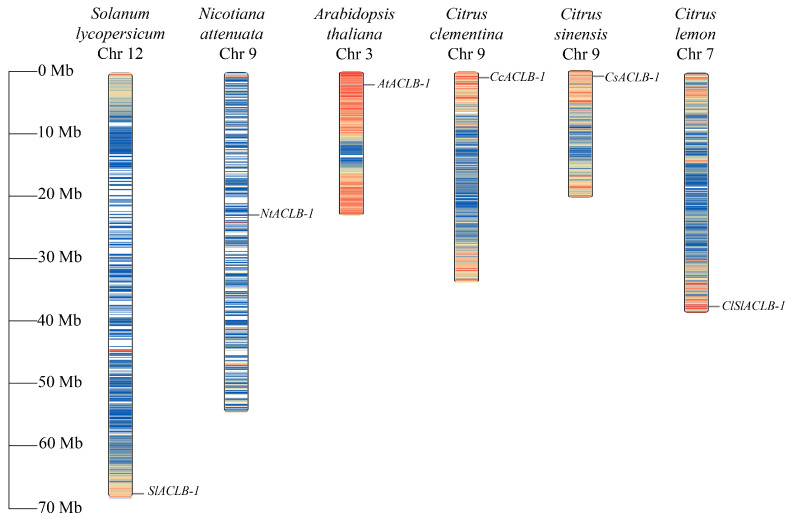
Homologous gene mapping of *ClACLB-1* and other species.

The *ClACLB-1* gene of ‘Xiangshui’ lemon is located on chromosome 7, while those of sweet orange and Clementine mandarin are both located on chromosome 9. In model plants, the *NtACLB*-1 gene is also located on chromosome 9, the *AtACLB-1* gene is located on chromosome 3, and the *SlACLB-1* gene is located on chromosome 12 (Figure 3).

### 2.4. Collinearity Analysis of the ClACLB-1 Gene

**Figure 4:**
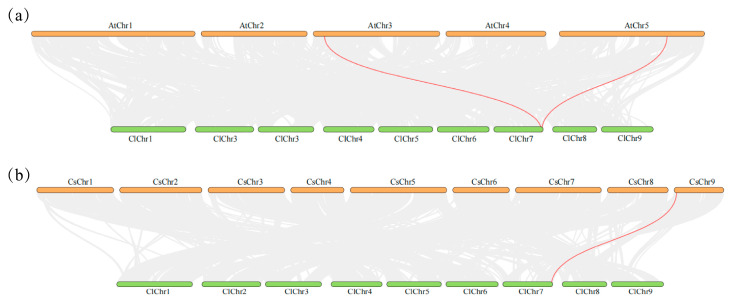
Collinearity analysis of *ClACLB-1* gene. (**a**) Interspecific collinearity analysis of *Citrus lemon* and *Arabidopsis thaliana*. (**b**) Interspecific collinearity analysis of *Citrus lemon* and *Citrus sinensis*.

The collinearity analysis results of the *ACLB-1* genes in *Citrus lemon* and *Arabidopsis thaliana* showed that there was one pair of homologous gene pairs of *ACLB-1* between the two species. The *ClACLB-1* gene had a collinear relationship with the homologous genes numbered AT3G06650.2 and AT5G49460.1 in *Arabidopsis thaliana* (Figure 4a). The collinearity analysis results of the *ACLB-1* genes in *Citrus lemon* and *Citrus sinensis* showed that there was one pair of *ACLB-1* homologous gene pairs between the two species. The *ClACLB-1* gene had a collinear relationship with the homologous gene numbered Cs9g02230.1 in *Citrus sinensis* (Figure 4b). This indicates that there is a gene segment duplication event of the *ACLB-1* gene in *Arabidopsis thaliana* but not in *Citrus lemon* and *Citrus sinensis*.

### 2.5. Analysis of Cis-Acting Elements in the Promoter of the ClACLB-1 Gene

**Figure 5:**
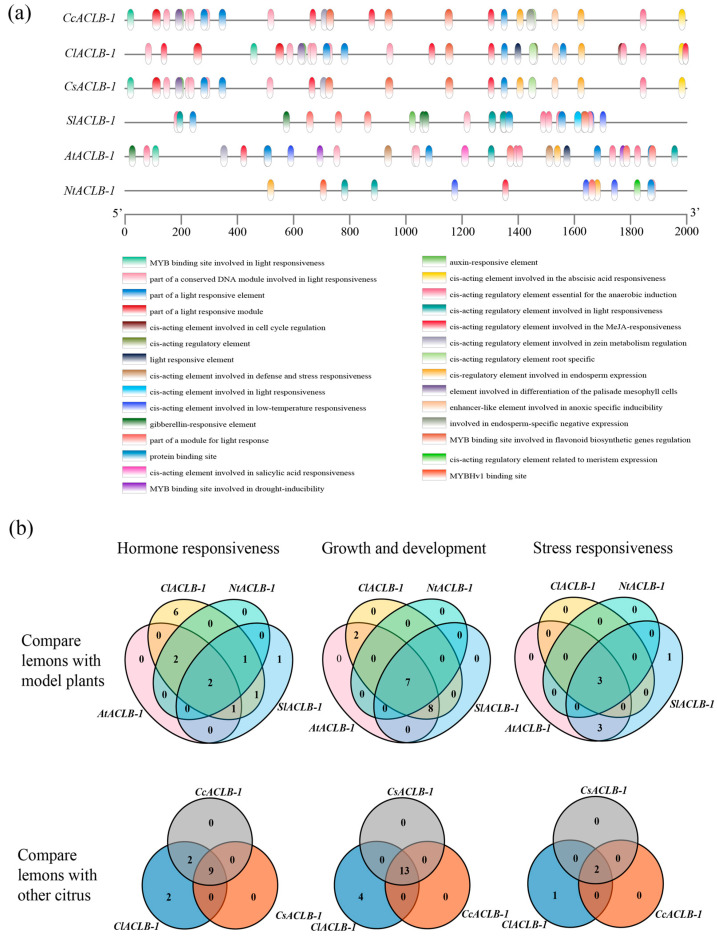
Analysis of cis-acting elements between *ClACLB-1* gene and homologous gene promoter of other five species. (**a**) The distribution of cis-acting elements in the promoter region. (**b**) Comparison of *ClACLB-1* gene of lemon with cis-acting elements of other species.

The PlantCARE database was utilized to predict potential cis-acting elements. Twenty-nine cis-acting elements were identified in the promoter regions of the *ACLB-1* genes of these six species in the promoter region of the gene (Figure 5a). These cis-acting elements were classified into three categories, namely hormone response elements, growth and development elements, and stress response elements. Among the hormone response elements, the *ClACLB-1* gene of lemon had the most elements. It shared nine elements with sweet orange and Clementine mandarin, and two elements with model plants. In the growth and development elements, light-responsive elements were the most numerous. The *ClACLB-1* gene of lemon had four more elements than sweet orange and Clementine mandarin and shared seven elements with model plants. In the stress response elements, the *ClACLB-1* gene of lemon had relatively fewer elements. It shared two elements with sweet orange and Clementine mandarin, while the *AtACLB-1* gene of *Arabidopsis thaliana* had the most elements (Figure 5b).

### 2.6. Analysis of the Subcellular Localization of the ClACLB-1 Protein

**Figure 6:**
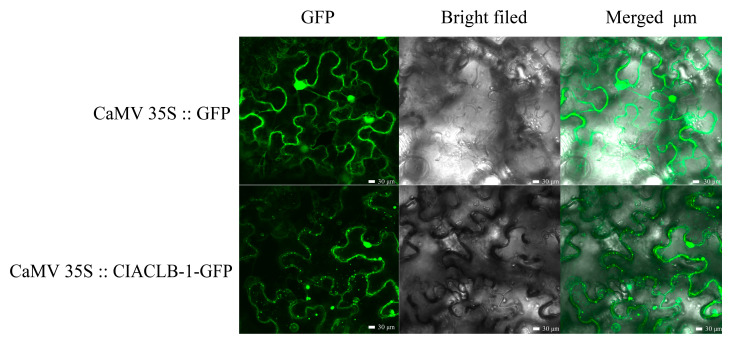
Subcellular localization analysis of ClACLB-1 protein of ‘Xiangshui’ lemon in Tobacco Ben (scale = 30 µm).

Through the transient transformation experiment on *Nicotiana benthamiana*, the results showed that the empty vector CaMV 35S::GFP could display green fluorescent signals in the cell membrane, nucleus and cytoplasm. Meanwhile, the fused protein CaMV 35S::ClACLB-1-GFP could also exhibit green fluorescence in the cell membrane and nucleus. This indicates that the subcellular localization of the ClACLB-1 protein is on the nucleus and cell membrane (Figure 6).

### 2.7. Analysis of the Spatiotemporal Expression Pattern of the ClACLB-1 Gene

**Figure 7:**
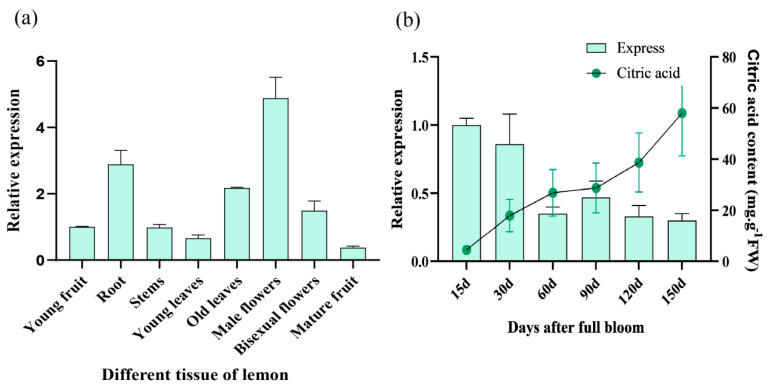
Analysis of spatiotemporal expression pattern of *ClACLB-1* gene. (**a**) Expression of *ClACLB-1* gene in different tissues. (**b**) Expression of *ClACLB-1* gene and changes in citric acid content during different fruit development stages.

The expression pattern of the *ClACLB-1* gene in different tissues of ‘Xiangshui’ lemon was explored. The results showed that the expression level of the *ClACLB-1* gene in the male flowers of ‘Xiangshui’ lemon was the highest, while the expression level was the lowest in the mature fruits (Figure 7a). During the fruit development process of ‘Xiangshui’ lemon, the content of citric acid gradually increased along with the development of the fruits. The expression level of the *ClACLB-1* gene in ‘Xiangshui’ lemon was the highest after 15 days of fruit setting. As the fruits developed, the expression level showed a fluctuating downward trend. At 60 and 90 days, during the middle stage of fruit development, the expression level increased to some extent but generally remained at a relatively low level. In general, the expression level of the *ClACLB-1* gene in the ‘Xiangshui’ lemon showed an overall downward trend, which was opposite to the changing trend of citric acid (Figure 7b).

### 2.8. The Citric Acid Content in Transgenic Tomatoes with the ClACLB-1 Gene Was Reduced

**Figure 8:**
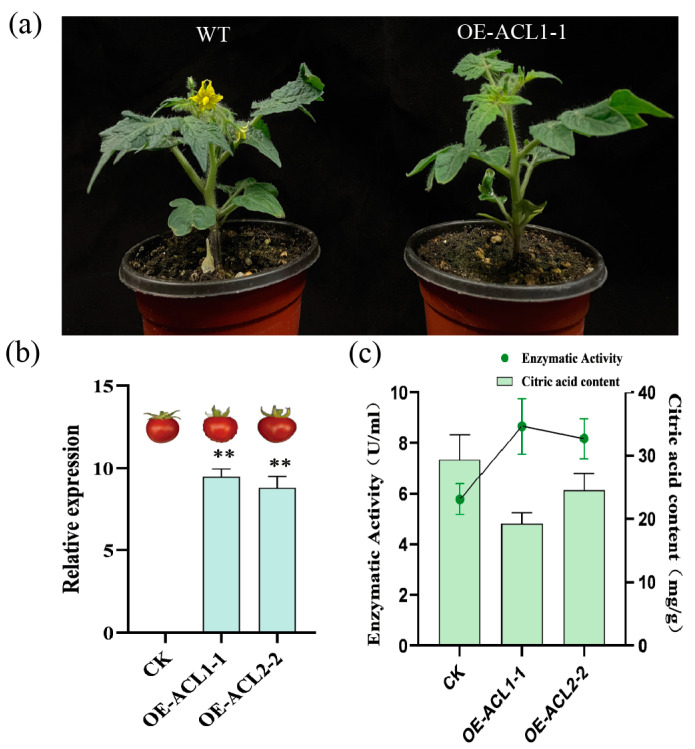
Phenotype and citric acid analysis of tomato plants overexpressing *ClACLB-1* gene. (**a**) Phenotype of transgenic tomato plants. (**b**) Expression of *ClACLB-1* gene in transgenic tomato. It was marked with ** is statistically different from the control test at 1% significance levels. (**c**) Enzyme activity and citric acid content in transgenic tomato.

In this experiment, three T0-generation tomato plants overexpressing the *ClACLB-1* gene were obtained, and eleven T2-generation positive plants were obtained. Among them, the plants numbered OE-ACL1-1 and OE-ACL2-2 had relatively high expression levels of the *ClACLB-1* gene, so their fruits were used for subsequent detection (Figure 8b). Compared with the control, the transgenic tomatoes overexpressing the *ClACLB-1* gene could all flower and bear fruits normally. There were slight differences in stem diameter, plant height and number of fruits, but they were not significant (Table 1, Figure 8a). Enzyme activity detection showed that the activity of ACL enzyme in the transgenic tomato fruits was significantly increased, and the content of citric acid was significantly decreased (Figure 8c).

### 2.9. The Overexpression of the ClACLB-1 Gene Led to an Increase in the Expression Level of the SlACLA-1 Gene

**Figure 9:**
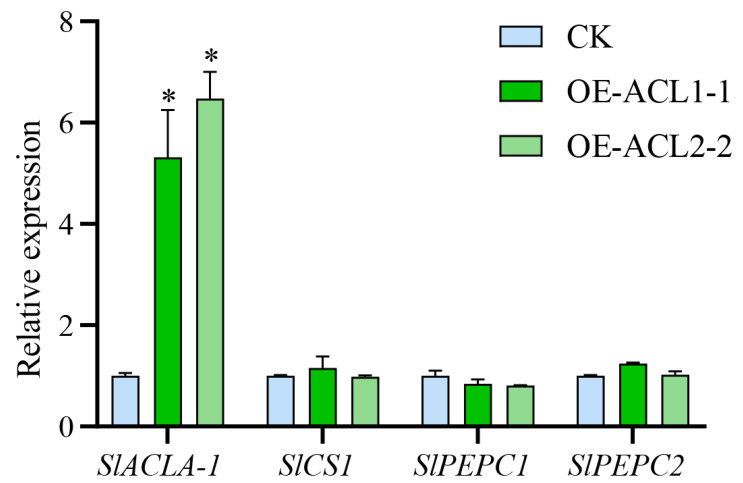
Expression of citric acid metabolism related genes in transgenic tomato. It was marked with * is statistically different from the control test at 5% significance levels.

The expression of endogenous genes related to citric acid metabolism was detected in transgenic tomato fruits. The results showed that the overexpression of the *ClACLB-1* gene could cause a significant increase in the expression level of the endogenous gene *SLACLA-1* in tomatoes. There were no significant changes in the expression of the citrate synthase gene *SLCS1* and the phosphoenolpyruvate carboxylase gene *SLPEPC2*, which are related to citric acid synthesis. The *SLPEPC1* gene showed a downward trend in expression, but the difference was not significant (Figure 9).

## 3. Discussion

Citric acid metabolism is a complex metabolic process in organisms. The citric acid cycle is an important metabolic process in organisms, which connects the metabolism of three major organic substances, involving numerous genes: carbohydrates, lipids, and proteins [26]. Studying the metabolism of citric acid in plants can provide theoretical reference for improving crop genes and plant stress resistance. The *ClACLB-1* gene in this study is a key gene involved in citric acid metabolism, and its functional research is important for agricultural research. The ACL pathway is one of the important pathways in citric acid metabolism. The gene related to ACL citrate lyase was first studied in 1992 [27]. In the past decade, the research on ACL citrate lyase was mainly focused on cotton [28], sugarcane [29], and citrus [24,30]. The most studied ones were mainly ACL in sweet orange and the three genes encoding ACL. The research directions mainly focused on functional verification, the influence of external environment, etc. Compared with previous studies, this article supplemented the collinearity analysis, phylogenetic tree analysis, and cis-acting element analysis of the *ACLB-1* gene and expounded the tissue and organ expression specificity in the spatiotemporal expression pattern in more detail. In transgenic tomatoes, it was explained that the reason for the decrease in citric acid was the increase in ACL enzyme activity.

The promoter is an important hub for controlling gene expression. It can accurately recognize the binding of RNA polymerase and initiate the transcription of DNA sequences [31]. Transcription factors can specifically interact with cis-acting elements in the promoter region of eukaryotic genes. Through direct or indirect interactions with other related proteins, they achieve regulatory functions of transcriptional activation or repression. In this study, 44.44% of the cis-acting elements of the *ClACLB-1* gene were identified as light-responsive elements, and stress-responsive elements accounted for only 8.3%. It was speculated that the lemon *ClACLB-1* gene plays a significant role in the process of plant growth and development and has an insignificant response to stress.

There are numerous experimental studies on the localization of ACL proteins. ACL is generally considered to be located in the cytoplasm, and a few studies also showed that ACL is located in chloroplasts and plastids [32,33]. In recent years, it was found that ACL exists in the nucleus [34]. This research suggested that ACL in the nucleus may enter through nuclear pores from the cytoplasm. The ClACLB-1 protein in this study was located on the nucleus and cell membrane. Some studies showed that the subcellular localization results of proteins are also related to the location of the interacting proteins [35]. It is speculated that this might be the reason why the ClACLB-1 protein is located on the cell membrane.

In the expression pattern in different tissues and organs, the *ClACLB-1* gene has the highest expression level in male flowers. Previous studies showed that the *ClACLB-1* gene also has the highest expression level in flowers in Anliu orange and Redanliu orange, but it does not indicate whether it is male flowers or bisexual flowers [24]. In addition, in transgenic tomatoes, the tomatoes overexpressing the *ClACLB-1* gene flower significantly later than the control. Does this imply that the *ClACLB-1* gene also has a certain function in regulating flowering? This question remains unanswered and is pending further research.

Previous studies verified the function of ACL in sweet oranges [24,30]. These studies were also conducted on transgenic tomatoes, and the experimental results were consistent with the results of this study, that is, the overexpression of the *ACLB-1* gene led to a decrease in citric acid. This study also showed the overexpression of the sweet orange *CsACLB-1* gene in sweet orange callus. The results showed that the activity of ACL enzyme increased, the citric acid also increased, which was contrary to the theoretical results. Another study explained that in the sweet orange callus overexpressing the *CsACLB-1* gene, the citric acid synthesis gene *CS1* also significantly increased [30]. However, in this study, the tomato *SlCS1* gene was detected, and no significant change was found, with only the expression level of the *ACLA-1* gene increasing significantly. It is speculated that the *ACLA-1* and *ACLB-1* genes have a synergistic effect. In other words, the overexpression of the *ACLB-1* gene will also lead to an increase in the expression level of the *ACLA-1* gene, resulting in an increase in ACL enzyme activity and a decrease in citric acid content.

## 4. Materials and Methods

### 4.1. Identification of ACLB-1 Genes

The whole-genome sequencing projects of ‘Xiangshui’ lemon were reported as in previous research [36]. The genomes of *Citrus sinensis*, *Citrus clementina*, *Arabidopsis thaliana*, *Nicotiana attenuate*, and *Solanum lycopersicum* were obtained from the website, http://citrus.hzau.edu.cn/download.php (accessed on 16 November 2023), and the website, https://plants.ensembl.org/info/data/ftp/index.html (accessed on 16 November 2023). Blast screening was performed to search the *ACLB-1* gene database of six species based on the sequences of *AtACLB-1* gene in *Arabidopsis thaliana* using TBtools software (V2. 138). The representative structural domain PF00285 was obtained from the website at http://pfam.xfam.org/ (accessed on 16 November 2023). Blast screening was performed to search the *ACLB-1* genes database of six species based on the domain PF00285. Two methods were combined to screen and obtain six species of *ACLB-1* genes.

### 4.2. Phylogenetic Analysis and Physicochemical Properties

Multiple-sequence alignment for these ACLB-1 proteins was performed utilizing the DNAman software (V6. 0). The phylogenetic analysis was conducted employing neighbor-joining (NJ). The final phylogenetic tree was then visually enhanced using the Interactive Tree of Life (iTOL) web tool, available at https://itol.embl.de/ (accessed on 16 November 2023). The physicochemical properties of *ClACLB-1* gene were predicted on Expasy PortParam, with the website found at https://web.expasy.org/protparam/ (accessed on 16 November 2023).

### 4.3. Prediction of Conservative Motifs, Conserved Domains, and Cis-Acting Elements

The conserved motifs of the *ACLB-1* genes in six species were performed and visualized using the MEME online tool. The conserved domains of *ACLB-1* genes were analyzed using the Conserved Domains Database (CDD) on the NCBI website, accessible at https://www.ncbi.nlm.nih.gov/Structure/bwrpsb/bwrpsb.cgi (accessed on 22 November 2023). The potential cis-acting regulatory elements within the *ACLB-1* genes of six species were scrutinized with the aid of the PlantCARE database. Finally, the results were visualized using TBtools software (V2. 138).

### 4.4. Chromosome Location and Collinearity Analysis

The chromosomal localization and visualization of *ACLB-1* genes of six species were performed using Tbtools software (V2. 138), and their collinearity analysis were also mapped with Tbtools software (V2. 138).

### 4.5. RNA Extraction and RT-qPCR Analysis

The samples of ‘Xiangshui’ lemon were collected from the individuals grown at Guangxi University, including the root, stems, male flowers, bisexual flowers, young fruit, mature fruit, young leaves, old leaves, and fruits at different developmental stages. The total RNA from the ‘Xiangshui’ lemon samples was extracted using the FastPure^®^ Plant Total RNA Isolation Kit (polysaccharides and polyphenolics-rich) (Vazyme, Nanjing, China). After checking the integrity of RNA, the AMV First Chain cDNA Synthesis Reagent (Sangong, Shanghai, China) was employed to reverse transcribe RNA into cDNA. The primers were designed using Primer 5.0 software, and the actin gene was used as a reference [37]; detailed information is provided in Table S1. The qRT-PCR experiment was conducted using the PerfectStart Green qPCR SuperMix (TansGen, Beijing, China). Finally, the relative expression levels were assessed using the 2^−ΔΔCt^ method [38].

### 4.6. Subcellular Localization

To perform subcellular localization analysis of ClACLB-1 proteins, the CDS of ClACLB-1 was amplified and cloned into the PEAQ-GFP vector to form ClACLB-1-GFP fusion proteins. The ClACLB-1-GFP fusion constructs were transfected into *Nicotiana benthamiana* leaves via *Agrobacterium tumefaciens* strain GV3101 transformation. After 3 d transfection, the GFP signals were captured using a confocal laser scanning microscope (TCS-SP8MP; Leica, Dusseldorf, Germany).

### 4.7. Verification of Transgenic Tomatoes

The seeds used in this experiment were Micro Tom, which were sourced from the Open Key Laboratory of Crop Genetic Improvement of the Guangxi Academy of Agricultural Sciences. In a super-clean bench, the seeds were disinfected with 75% alcohol for 30 s, rinsed with sterile water for 5–8 times, with each rinse lasting 2 min. For the last rinse, they were soaked in sterile water for 30 min. Then, the moisture was absorbed with sterilized toilet paper, and the seeds were sown in the T0 medium. After 7–10 days, the hypocotyls of tomato cotyledons were taken and cut into 5 × 5 square pieces. They were placed face up in the pre-culture T1 medium. The medium was wrapped with tin foil and placed in a constant temperature environment of 26 °C for dark culture for 2 days. After 2 days, they were taken out, and the tomato cotyledons were placed in the infection solution. They were inverted and mixed by shaking for 30 min. The bacterial solution was absorbed with sterile paper, and the cotyledons were placed with the back of the leaves facing up on the T2 screening medium. It should be noted that continuous observation was required within 7 days. If there was bacterial contamination, the medium should be replaced in time. After being placed in the T2 medium for 25–30 days, newly emerged tomato buds could be observed. In the super-clean bench, the buds were cut off with a scalpel and placed in the T3 rooting medium. It should be noted that the rooting medium needed to be prepared and used immediately, as a long preparation time would cause the medium to become ineffective. When the newly emerged plants grew to a height of 5 cm with well-developed roots, they could be transplanted into the soil.

To prepare the infection solution, 100 µL of positive Agrobacterium was taken and put it into 1.5 mL of LB liquid medium (resistant to kanamycin and rifampicin) and cultured overnight at a constant temperature of 28 °C. On the second day, 300 µL of the bacterial solution was taken and put it into 20 mL of LB liquid medium (resistant to kanamycin and rifampicin) and expanded and shaken at a constant temperature of 28 °C for 6–7 h. After the expansion and shaking, the bacterial solution was taken out and centrifuged at 5000 rpm for 10 min; then, the supernatant was poured out, and the LB liquid medium without resistance was added again; the centrifugation and pouring out of the supernatant was repeated. Finally, the bacterial solution was diluted with MS liquid medium until the OD_600_ value was between 0.1 and 0.3. If the subsequent medium was still likely to be contaminated by Agrobacterium, the OD_600_ value of the diluted bacterial solution could be adjusted to below 0.1.

### 4.8. Detection of ACL Citrate Lyase Activity

The activity of the enzyme was measured using the Plant ATP Citrate Lyase ELISA Kit Instruction (Thermo Fisher, Shanghai, China). The specific method is as follows: Take 0.1 g of plant sample powder and dissolve it in 1 mL of sterile water. Firstly, take the kit samples out of the refrigerator and allow them to equilibrate to room temperature for 20 min. Next, set up the standard wells and sample wells on the microplate. The concentrations of the standards are 0, 1, 2, 4, 8, and 16 U/mL, respectively. Add the samples to be tested into the sample wells, then add the sample diluent, and add the HRP-labeled detection antibody to each well. Use a plate sealing film to cover the reaction wells, and then incubate in a water bath or an incubator at 37 °C for 60 min. After that, discard the liquid, absorb the liquid in each well with absorbent paper, fill each well with the washing solution, let it stand for 1 min, and then remove the washing solution and absorb the liquid again. Repeat the plate washing operation 5 times, then add 50 µL each of substrate A and substrate B, and incubate at 37 °C in the dark for 15 min. Finally, add 50 µL of the stop solution, and measure the OD value of each well at a wavelength of 450 nm within 15 min.

### 4.9. Determination of Fruit Quality

Prepare the standard stock solutions of malic acid, tartaric acid, and citric acid at a concentration of 1.0 mg/mL, respectively. Using the standard stock solutions as the mother liquor, prepare the mixed standard solutions with the concentration gradients of 5.0, 10.0, 20.0, 30.0, 40.0, and 50.0 mg/L. In the liquid chromatograph, inject 25 µL samples in sequence for measurement of the peak area. With the concentration of organic acids as the abscissa and the peak area as the ordinate, the standard curves of the three organic acids can be obtained. Weigh 5.0 g of lemon samples, and freeze and grind them to prepare the sample extract. After diluting it 50 times with ultrapure water, filter it through 0.22 µm microporous membrane. Measure it on the instrument to obtain different peak areas. Substitute the peak areas into the standard curves to calculate the content of each organic acid in the fruit samples [39].

## 5. Conclusions

In this study, the *ClACLB-1* gene was mined from the genome of ‘Xiangshui’ lemon. Transgenic tomato experiments demonstrated that the overexpression of the *ClACLB-1* gene could cause a significant increase in the *ACLA-1* gene, thereby enhancing ACL enzyme activity, cleaving citric acid, and leading to a significant decrease in the citric acid content in the fruits.

## Figures and Tables

**Table 1:** Phenotypic analysis of T2 transgenic Micro Tom.

Number	Stem Thickness (mm)	Plant Height (mm)	Number of Fruits	First Flowering Time	Fruit Development Period (Days)
WT	4.29 ± 0.16 a	79.42 ± 0.13 a	9	25 December	26.00 ± 3.00 a
OE-ACL1-1	4.03 ± 0.11 a	83.56 ± 0.17 a	9	7 January	26.67 ± 0.58 a
OE-ACL2-2	4.36 ± 0.11 a	82.61 ± 2.66 a	6	4 January	27.33 ± 4.93 a

Note: The letter ‘a’ means they are not statistically significant

## Data Availability

The raw data supporting the conclusions of this article will be made available by the authors upon request.

## Supplementary Materials

The following supporting information can be downloaded at: https://www.mdpi.com/article/10.3390/plants14010053/s1. Table S1. Primers used for RT-qPCR.

## References

[1] Gironés-Vilaplana A., Valentão P., Andrade P.B., Ferreres F., Moreno D.A., García-Viguera C. (2015). Beverages of lemon juice and exotic noni and papaya with potential for anticholinergic effects. Food Chem..

[2] Zhou S.Q., Sakamoto K. (2020). Citric acid promoted melanin synthesis in B16F10 mouse melanoma cells, but inhibited it in human epidermal melanocytes and HMV-II melanoma cells via the GSK3β/β-catenin signaling pathway. PLoS ONE.

[3] Hu X.Z. (2003). How vinegar and citric acid perform health functions. Capit. Food Med..

[4] Jia Y.Q., Zhao M.X., Zhao L., Xu Y.L., Zhang Q.L., Li J.J., Xu F.M. (2024). Research progress on citric acid and its derivatives in the prevention and treatment of renal calcium oxalate stones. West China J. Pharm. Sci..

[5] Bi T.T., Wu G.H., Wang Y.Y., Tang Y.M., Liu L. (2023). Development of bamboo shoot and hawthorn beverage. Farm Prod. Process..

[6] Wei Z., Chen Y.H., Li X.Q., Rong H.Y., Huang Z.J. (2022). Remediation of heavy metal contaminated farmland soil by biodegradable chelating agent GLDA. Appl. Sci..

[7] Li W.H., Feng Y.X., Hu Z.R., Yi C., Zhu H.Y., Xu G.Y., Cao Y.E., Li W.H. (2024). Impacts of organic materials combined with citric acid on soil characteristics, strawberry quality and yield. J. Jiangsu Agric. Sci..

[8] Yi X., Wu N., Li J.Y., Wang H.B., Zhang L. (2024). Effects of UV-C and citric acid treatment on storage quality of postharvest banana. Chin. J. Trop. Crops.

[9] Massoud M.B., Sakouhi L., Chaoui A. (2019). Effect of plant growth regulators, calcium and citric acid on copper toxicity in pea seedlings. J. Plant Nutr..

[10] Sehar A., Shafaqat A., Aslam B.S., Muhammad R., Mujahid F., Farhat A., Muhammad I., Aamer M.M., Hasan G.A. (2015). Citric acid enhances the phytoextraction of chromium, plant growth, and photosynthesis by alleviating the oxidative damages in *Brassica napus* L.. Environ. Sci. Pollut. Res. Int..

[11] Bilal H.S., Shi C.Y., Guo L.X., Muhammad K.H., Avi S., Liu Y.Z. (2017). Recent advances in the regulation of citric acid metabolism in citrus fruit. Crit. Rev. Plant Sci..

[12] Sadka A., Dahan E., Cohen L., Marsh K.B. (2003). Aconitase activity and expression during the development of lemon fruit. Physiol. Plant..

[13] Ratajczak R. (2000). Structure, function and regulation of the plant vacuolar H^+^-translocating ATPase. Biochim. Biophys. Acta.

[14] Maeshima M. (2000). Vacuolar H+-pyrophosphatase. Biochim. Biophys. Acta.

[15] Sadka A., Dahan E., Or E., Roose M.L., Marsh K.B., Cohen L. (2000). Comparative analysis of mitochondrial citrate synthase gene structure, transcript level and enzymatic activity in acidless and acid-containing citrus varieties. Funct. Plant Biol..

[16] Etienne A., Génard M., Lobit P., Mbeguié-A-Mbéguié D., Bugaud C. (2013). What controls fleshy fruit acidity a review of malate and citrate accumulation in fruit cells. J. Exp. Bot..

[17] Burke A.C., Huff M.W. (2017). ATP-citrate lyase: Genetics, molecular biology and therapeutic target for dyslipidemia. Curr. Opin. Lipidol..

[18] Cercós M., Soler G., Iglesias D.J., Gadea J., Forment J., Talón M. (2006). Global analysis of gene expression during development and ripening of citrus fruit flesh: A proposed mechanism for citric acid utilization. Plant Mol. Biol..

[19] Asfaw D., Bayissa H., Adriano N.N., Ludmila S., Naftali Z., Ehud K., Fernie A.R., Eduardo B., Avi S. (2011). Inhibition of aconitase in citrus fruit callus results in a metabolic shift towards amino acid biosynthesis. Planta.

[20] Katz E., Boo K.H., Kim H.Y., Eigenheer R.A., Phinney B.S., Shulaev V., Negre-Zakharov F., Sadka A., Blumwald E. (2011). Label-free shotgun proteomics and metabolite analysis reveal a significant metabolic shift during citrus fruit development. J. Exp. Bot..

[21] Aprile A., Federici C., Close T.J., De B.L., Cattivelli L., Roose M.L. (2011). Expression of the H+-ATPase AHA10 proton pump is associated with citric acid accumulation in lemon juice sac cells. Funct. Integr. Genom..

[22] Chen M., Xie X.L., Lin Q., Chen J.Y., Grierson D., Yin X.R., Sun C.D., Chen K.S. (2013). Differential expression of organic acid degradation-related genes during firuit development of navel oranges (*Citrus sinensis*) in two habitats. Plant Mol. Biol. Rep..

[23] Li S.J., Wang W.L., Ma Y.C., Liu S.C., Grierson D., Yin X.R., Chen K.S. (2020). Citrus *CitERF6* contributes to citric acid degradation via upregulation of *CitAcl* alpha1, encoding ATP-Citrate Lyase subunit alpha. J. Agric. Food Chem..

[24] Hu X.M., Shi C.Y., Liu X., Jin L.F., Liu Y.Z., Peng S.A. (2015). Genome-wide identification of citrus ATP-citrate lyase genes and their transcript analysis in fruits reveals their possible role in citrate utilization. Mol. Genet. Genom..

[25] Guo L.X., Shi C.Y., Liu X., Ning D.Y., Jing L.F., Yang H., Liu Y.Z. (2016). Citrate accumulation-related gene expression and/or enzyme activity analysis combined with metabolomics provide a novel insight for an orange mutant. Sci. Rep..

[26] Zhang H.Y., Hou J., Yang X.Z., Li J., Zeng K.F., Yao S.X., Zhang H.Y. (2023). Study on citric acid degradation pathways of blood orange during segment drying based on widely targeted metabolomics. J. Food Sci. Technol..

[27] Elshourbagy N.A., Ncar J.C., Kmetz P.J., Sathe G.M., Southan C., Strickler J.E., Gross M., Yong J.F., Wells T.N., Groot P.H. (1990). Rat ATP citrate-lyase. Molecular cloning and sequence analysis of a full-length cDNA and mRNA abundance as a function of diet, organ, and age. J. Biol. Chem..

[28] Liu F.J., Ma Z.F., Cai S., Dai L.J., Gao J.B., Zhou B.L. (2022). ATP-citrate lyase B (*ACLB*) negatively affects cell death and resistance to Verticillium wilt. BMC Plant Biol..

[29] Phan T.T., Li J., Sun B., Liu J.Y., Zhao W.H., Huang C., Yang L.T., Li Y.R. (2017). ATP-citrate lyase gene (*SoACLA-1*), a novel *ACLA* Gene in dugarcane, and its overexpression enhance drought tolerance of transgenic tobacco. Sugar Tech.

[30] Guo L.X. (2020). Studying the Role of Citrus ATP-Citrate Lyase (ACL) in the Accumulation of Citrate and Its Relative Metabolites, as Well as ACL Action Mechanism and AC-Affecting Factors. Ph.D. Thesis.

[31] Sri T., Gupta B., Tyagi S., Singh A. (2020). Homeologs of Brassica *SOC1*, a central regulator of flowering time, are differentially regulated due to partitioning of evolutionarily conserved transcription factor binding sites in promoters. Mol. Phylogenet. Evol..

[32] Rangasamy D., Ratledge C. (2000). Compartmentation of ATP-citrate lyase in plants. Plant Physiol..

[33] Ma Y., Jakowitsch J., Maier T., Bayer M., Müller N., Schenk H., Loffelhardt W. (2001). ATP citrate lyase in the glaucocystophyte alga Cyanophora paradoxa is a cytosolic enzyme: Characterisation of the gene for the large subunit at the cDNA and genomic levels. Mol. Genet. Genom..

[34] Shen Y., Wei W., Zhou D.X. (2015). Histone acetylation enzymes coordinate metabolism andgene expression. Trends Plant Sci..

[35] Chang S., Simon W., Melissa D., Mark R. (2009). Protein-protein interaction as a predictor of subcellular location. BMC Syst. Biol..

[36] Yu H., Zhang C., Lu C., Wang Y.N., Ge C.C., Huang G.X., Wang H.F. (2024). The lemon genome and DNA methylome unveil epigenetic regulation of citric acid biosynthesis during fruit development. Hortic. Res..

[37] Patrick W.J., Botha F.C., Birchi R.G. (2012). Metabolic engineering of sugars and simple sugar derivatives in plants. Plant Biotechnol. J..

[38] Livak K.J., Schmittgen T.D. (2001). Analysis of relative gene expression data using real-time quantitative PCR and the 2^−ΔΔCT^ method. Methods.

[39] Wang Y.N., Lu C., Yang Y.L., Tang T., Yan X.Y., Huang G.X. (2023). Identification and expression analysis of key enzyme gene family in lemon sucrose metabolism. J. S. Agric..

