# Supplementary material for: ATP Citrate Lyase ClACLB-1 Facilitates Citrate Cleavage in Lemon
Source: Plants (Basel). 2024 Dec 27;14(1):53. doi: 10.3390/plants14010053 (PMC11722762; doi:10.3390/plants14010053)
Supplement: Supplementary file 1 [file plants-14-00053-s001.zip › plants-3381991-supplementary.pdf]

Table S1. Primers used for RT-qPCR

Gene ID	Forward primer	Reverse primer sequence
ACTB	CCAATTCTCTCTTGAACCTGTCCTT	TGACTGATGAGAACTGCCAGAAG
ClACLB-1	AACAGGGAAAGGTAAACAAAC	AGCAGACTCCATCTCACCACC
SLACTIN	GAGGATATTCAGCCCCTTGTTTG	CATCTTTCTGACCCATTCCAACC
SlACLA1	ATTGTGGTTCAAACGCAGCC	CCTGGCAGTTACGATGGTGT
SlCS1	TTGGGGAACATCACAGTTG	TGATGGCACCTTTCCTGTT
SlPEPC1	TGTGAACCTGAACCCGACT	GTCCCCTATTCGGGACTTC
SlPEPC2	TATCACTACTTTAAATGTCTGC	TCAAGGATACATGATTCTTAAT
